# Body Image and Other Mood Vulnerabilities in Adolescents with Polycystic Ovary Syndrome and Metabolic Alterations

**DOI:** 10.3390/children11050521

**Published:** 2024-04-26

**Authors:** Federica Barbagallo, Lara Tiranini, Chiara Placentino, Giacomo Mariacci, Manuela Piccinino, Laura Cucinella, Aldo E. Calogero, Rossella E. Nappi

**Affiliations:** 1 Department of Clinical and Experimental Medicine, University of Catania, 95124 Catania, Italy; federica.barbagallo@phd.unict.it (F.B.); acaloger@unict.it (A.E.C.); 2 Department of Clinical, Surgical, Diagnostic and Pediatric Sciences, University of Pavia, 27100 Pavia, Italy; lara.tiranini01@universitadipavia.it (L.T.); chiara.placentino01@universitadipavia.it (C.P.); giacomo.mariacci01@universitadipavia.it (G.M.); laura.cucinella01@universitadipavia.it (L.C.); 3 Research Center for Reproductive Medicine, Gynecological Endocrinology and Menopause, IRCCS San Matteo Foundation, 27100 Pavia, Italy; m.piccinino@smatteo.pv.it

**Keywords:** polycystic ovary syndrome (PCOS), adolescence, obesity, insulin resistance, body image, anxiety, depression, eating disorders

## Abstract

Introduction: Psychological vulnerability is a relevant component of polycystic ovarian syndrome (PCOS), but it is still under-explored, especially during adolescence. The aim of this study was to describe a selection of psychometric characteristics in a clinical sample of Italian adolescents with PCOS. Moreover, we reported the associations of body image, eating attitudes, and mood with metabolic features. Methods: Our sample included 128 adolescent girls (age range: 14–19 years) with PCOS. Validated psychometric questionnaires were administered: State Anxiety Inventory (STAI), Beck Depression Inventory (BDI), Body Attitude Test (BAT), Bulimia Investigation Test (BITE), Eating Attitudes Test (EAT), and Perceived Stress Scale (PSS). Results: Anxiety was the most prevalent mood disorder (63.1% trait anxiety and 57% state anxiety). Our cohort also showed a high prevalence of depression (39.1%), body image dissatisfaction (49.2%), disordered eating (11.7%), and bulimic risk (41.4%). PCOS adolescents with obesity and insulin resistance (IR) had statistically significant higher body image distress compared to those with normal weight and without IR (*p* < 0.001). The Sobel test for mediation showed that body image dissatisfaction mediates the relationship between state anxiety and bulimic risk (Z = 3.42, *p* < 0.001) and between depression and bulimic risk (Z = 4.59, *p* < 0.001). Conclusions: A considerable number of patients with PCOS experience psychological disorders during adolescence. IR and obesity play a role in the distress associated with body image, further contributing to psychological vulnerability, especially in the bulimic domain. A comprehensive biopsychosocial approach in adolescents with PCOS represents the basis for effectively managing and preventing complications arising from both psychological and biological disorders in adulthood.

## 1. Introduction

Polycystic ovary syndrome (PCOS) is one of the most prevalent endocrine disorders, affecting between 10 and 13% of women of reproductive age [1] and 6–8% of adolescent girls [2]. According to the World Health Organization, adolescence is the phase of life between 10 and 19 years. It requires special care, representing an important time to lay the foundations of good health in adulthood and for future generations [3].

The clinical presentation of PCOS is heterogeneous and varies across the lifespan, from adolescence to menopause, within and between women. Despite the misnomer that defines this disease, PCOS is not a problem of ovarian microcysts but a complex disorder characterized by reproductive, metabolic, and psychosocial features [1,4].

The diagnosis of PCOS is controversial, especially in adolescence, because many characteristics used to diagnose PCOS in adulthood (acne, irregular menses, and polycystic ovary morphology) may be physiological during the peripubertal period [2]. According to the Rotterdam criteria, at least two of the following three criteria are needed to diagnose PCOS: (1) menstrual irregularity and/or ovulatory dysfunction (OD); (2) clinical and/or biochemical hyperandrogenism; (3) polycystic ovary morphology (PCOM) [5]. However, the latest recent international evidence-based guideline [1] states that pelvic ultrasound is not recommended for the diagnosis of PCOS during adolescence, due to the high rate of multifollicular ovaries (MFOs) in the early years of reproductive life [1]. Therefore, both hyperandrogenism and ovulatory dysfunction are required to diagnose PCOS in adolescent girls [1]. For adolescents presenting isolated symptoms, such as irregular menstrual cycles or hyperandrogenism, a reevaluation may be considered after full reproductive maturity (more than 8 years after menarche) [1]. 

Although the diagnostic criteria of PCOS do not include the dysmetabolic component, it is widely recognized that obesity, insulin resistance (IR), and hyperinsulinemia are commonly part of this condition [6]. Up to 80% of PCOS women exhibit IR, which is independent of body mass index (BMI) and plays an important role in the clinical presentation and development of several metabolic alterations [7]. In adult women diagnosed with PCOS, BMI has been reported to correlate with poor quality of life (QoL), not only directly but also indirectly through the mediation of dysmorphic concerns [8]. Previous studies have shown that BMI is a predictor of body dissatisfaction in young adolescent girls [9]. Furthermore, body image concerns seem to be among the most important components in developing and maintaining eating disorders, which are often associated with PCOS [10], along with mild to moderate mental health problems. However, despite the higher prevalence of anxiety and depression compared to controls (young girls without PCOS) [11], emotional well-being is still a poorly assessed area in PCOS, especially during adolescence. 

The aim of the present study is to describe a selection of psychometric characteristics in a clinical sample of Italian adolescent girls with PCOS. Moreover, we report the associations of body image, eating attitudes, mood, and perceived stress with weight and metabolic features.

## 2. Materials and Methods

### 2.1. The Cohort

This is a retrospective study conducted on 128 adolescents with PCOS referred to the Unit of Gynecological Endocrinology, IRCCS San Matteo Foundation, University of Pavia (Pavia, Italy), in North-West Italy. This study was conducted according to the Ethical Guidelines of the Declaration of Helsinki, and the local ethics committee approved the analysis of medical records of patients who signed informed consent (University of Pavia—IRCCS San Matteo RC-14-24). During the consultation conducted by a gynecologist expert in endocrinology, a thorough medical history was obtained from each patient, and their general health status, family history, and previous and current use of medications were recorded. When needed, clinical interview was completed with the help of a significant other (e.g., mother, older sister). Physical examination included measurement of body weight and height, and the body mass index (BMI) was calculated. Waist circumference was measured at the horizontal plane, midway between the lowest ribs and the iliac crest. Hip circumference was measured at the level of the largest lateral extension of the hips. Blood pressure was measured by supporting the upper limb, ensuring the cuff was at heart level. PCOS was diagnosed according to the latest recent international evidence-based guideline reported above [1]. A comprehensive history and physical examination were performed to assess symptoms and signs of clinical hyperandrogenism, including severe acne and hirsutism. Since pelvic ultrasound is not recommended for the diagnosis of PCOS during adolescence, both clinical hyperandrogenism and menstrual irregularities were present in our cohort. Exclusion criteria included the following: pregnancy and lactation, any hormonal treatment in the last three months, major endocrine and psychiatric disorders, psychoactive medications, illicit drug use, and smoking habits. 

Hormonal measurements included the following: follicle-stimulating hormone (FSH) (follicular phase range 2.8–11.3 IU/L), luteinizing hormone (LH) (follicular phase range 1.1–11.6 IU/L), 17β-estradiol (E_2_) (follicular phase range 15–160 pg/mL), androstenedione (normal range 0.46–3.39 ng/mL), total testosterone (TT) (normal range: 0.15–0.80 ng/mL), dehydroepiandrosterone sulfate (DHEAS) (normal range 0.35–4.3 mcg/mL), and insulin (normal range 3–25 mIU/L). FSH, LH, E_2_, androstenedione, TT, and DHEAS were measured by chemiluminescence (SIEMENS Immulite 2000, Munich, Germany). Information available on routine metabolic parameters includes the following: fasting glucose (mg/dL), total cholesterol (mg/dL), high-density lipoprotein (HDL) (mg/dL), triglycerides (TGL) (mg/dL), aspartate aminotransferase (AST) (mU/mL), and alanine aminotransferase (ALT) (mU/mL). We calculated the level of low-density lipoprotein (LDL) to complete the framework for the glycolipid profile. The homeostasis model assessment (HOMA) index was calculated for the evaluation of IR using the formula [glycemia (mg/dL) × insulin (µIU/mL)]/405, and the values in the 0.23–2.5 range were considered normal. The visceral adiposity index (VAI) was calculated using the equation waist circumference (WC)/[36.58 + (1.89 × BMI)] × (TGL/0.81) × (1.52/HDL-C) [12]. The VAI has been described as a valuable indicator of “visceral adipose function” and insulin sensitivity, and its increase has been strongly associated with cardiometabolic risk [12].

### 2.2. Psychometric Questionnaires

Patients were asked to fill in a set of validated psychometric questionnaires to evaluate body image, eating attitudes, mood, and perceived stress. It is standard routine for our unit to collect information on the psychological well-being of outpatients with menstrual dysfunction. 

#### 2.2.1. Body Image

The Body Attitude Test (BAT) was designed to evaluate body image disturbance. It consists of 20 items, grouped into four main factors: negative appreciation of body size, lack of familiarity with one’s own body, general body dissatisfaction, and rest factor. Each item is assigned a maximum of 6 points (from 0 to 5). A value of 36 represents the cut-off value for distinguishing between the clinical population and the general population [13].

#### 2.2.2. Eating Disorders

The Bulimia Investigation Test (BITE) [14] was used to investigate bulimic risk. The questionnaire investigates habits of dieting and symptoms and behaviors associated with binge eating. According to the total score, patients are classified as low risk (<10), medium risk (10–24), and high risk (≥25) of having bulimia.

The Eating Attitudes Test (EAT) is a 26-item self-report questionnaire designed to assess eating disorder symptomatology. The total score (between 0 and 78) provides an overall risk score, with higher scores indicating a greater risk of an eating disorder. Total scores of 20 or greater are considered to be in the clinical range [15].

#### 2.2.3. Mood

The State Anxiety Inventory (STAI) was used to evaluate two types of anxiety symptoms: state (i.e., how a person in the current situation responds to perceived threat) and trait anxiety (i.e., the stable tendency to attend, experience, and report negative emotions). A cut-off of 40 was used to evaluate the presence or absence of state and trait anxiety [16]. 

The Beck Depression Inventory (BDI) is a self-reported questionnaire consisting of 21 items, which evaluate the severity of depressive symptoms. The total score ranges from 0 to 63: <10 = no depression; 10–18 = mild; 19–29 = moderate; and 30–63 = severe [17]. 

#### 2.2.4. Perceived Stress

The Perceived Stress Scale (PSS-10) is a 10-item questionnaire [18], widely used to assess stress levels in youth and adults aged 12 and older. It assesses the degree to which an individual perceives life as unpredictable, uncontrollable, and overloading in the previous month. Higher scores indicate greater levels of perceived stress. Participants were classified as women with low (0–6), moderate (17–19), high (20–25), very high (>25) perceived stress. 

### 2.3. Statistical Analysis

Descriptive statistics with frequency distributions were used to study the prevalence of mental health morbidity. The Kolmogorov–Smirnov test was used to evaluate whether each variable had a Gaussian distribution. Student’s *t*-test and Mann–Whitney U test were used for continuous variables, where appropriate. Spearman correlation analysis was used to investigate the association between body image, clinical characteristics, and metabolic and hormonal profiles. Patients were also divided into four subgroups based on BMI and IR: (1) normal weight without IR (BMI < 25 and HOMA index < 2.5) (*n* = 73); (2) overweight without IR (BMI ≥ 25 and HOMA index < 2.5) (*n* = 27); (3) normal weight with IR (BMI < 25 and HOMA index ≥ 2.5) (*n* = 5); and overweight with IR (BMI ≥ 25 and HOMA index ≥ 2.5) (*n* = 23). Finally, we tested the mediation effect between anxiety (STAI-I), depression (BDI), body image dissatisfaction (BAT), and bulimic risk (BITE) using the Sobel test for mediation [19]. The independent variable was the BITE score. The dependent variables were STAI-1 and BDI scores, and the posited mediator was BAT total score. SPSS 23.0 software for Windows (SPSS Inc., Chicago, IL, USA) was used for data analysis, and a *p*-value less than 0.05 was considered statistically significant.

## 3. Results

The mean age of the enrolled patients was 17.2 years (standard deviation (SD): 1.6 years), with an age range of 14 and 19 years. The demographic, clinical, and metabolic characteristics of the enrolled patients are reported in Table 1. 

Anxiety was the most prevalent mood disorder in this cohort. We found that 63.1% of the patients (*n* = 81) had trait anxiety, and 57% (*n* = 73) had a clinical state of anxiety. Trait anxiety was mild in 32.8% of patients (*n* = 42), moderate in 16.4% (*n* = 21), and severe in 14.1% (*n* = 18). A mild clinical state of anxiety was present in 29.7% (*n* = 38), whereas there was moderate and severe anxiety in 18% (*n* = 23) and 9.4% (*n* = 12), respectively. Depression was present in 39.1% (*n* = 50) of the study sample: 25.8% of patients had mild depression (*n* = 33), 9.4% moderate depression (*n* = 12), and only 3.9% severe depression (*n* = 5). A high prevalence of body image dissatisfaction (49.2%, *n* = 63), eating abnormalities (11.7%, *n* = 15), and bulimic risk (41.4%, *n* = 53) was also shown, and 68.2% (*n* = 60/88) of the cohort reported high and very high scores of perceived stress. Table 2 shows the psychometric characteristics in adolescent girls with PCOS. 

**Figure 1:**
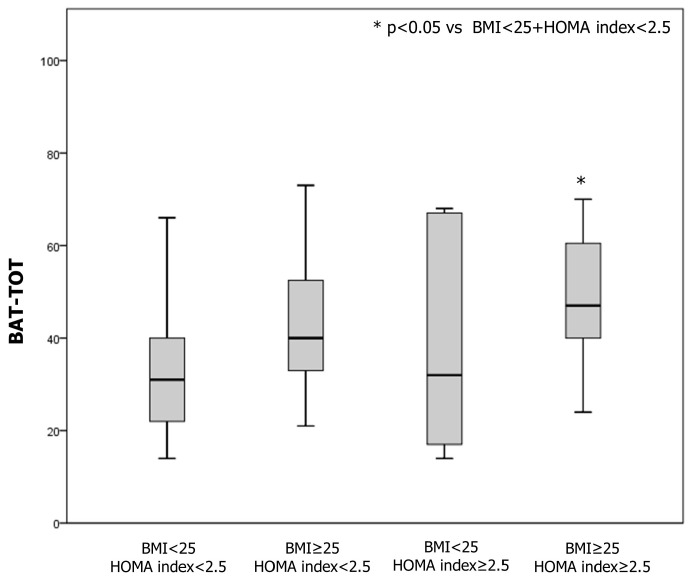
Comparison of body attitude distress evaluated by body attitude test (BAT) in adolescents with polycystic ovary syndrome (PCOS) according to body mass index (BMI) and insulin resistance.

Patients were also divided into four subgroups based on BMI and IR: (1) normal weight without IR (BMI < 25 and HOMA index < 2.5); (2) overweight without IR (BMI ≥ 25 and HOMA index < 2.5); (3) normal weight with IR (BMI < 25 and HOMA index ≥ 2.5); and overweight with IR (BMI ≥ 25 and HOMA index ≥ 2.5). A statistically significant difference in body image distress was found in these subgroups (Figure 1 and Table 3). No statistically significant differences were found between androgen levels in patients with IR and without IR, and by BMI. Patients with overweight and IR also showed poorer scores in the following subgroups of the BAT compared to patients without obesity and IR: “negative appreciation of body size”, “lack of familiarity with one’s own body”, and “general body dissatisfaction” (Supplementary Table S1). 

No significant differences were found in anxiety, depression, stress, and eating disorders among PCOS adolescents based on their BMI (Table 3). Adolescents with PCOS and IR showed statistically significantly higher scores of STAI-I for state anxiety and of PSS for perceived stress compared to PCOS adolescents without IR. 

**Figure 2:**
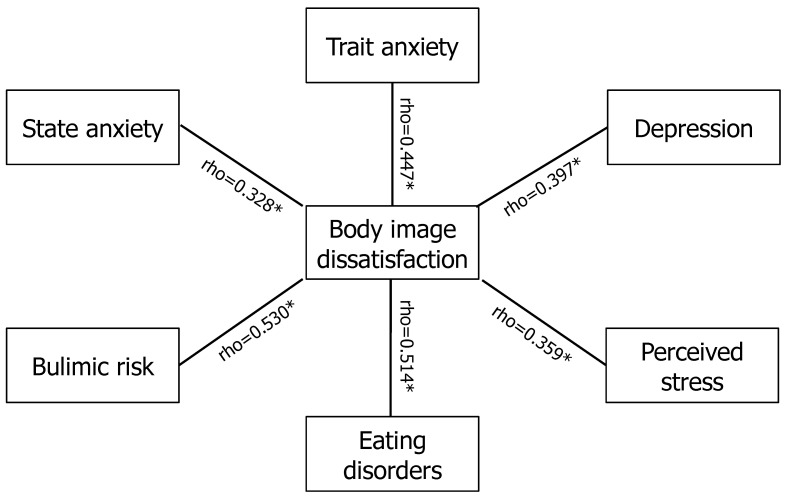
Illustration of Spearman correlation between body image dissatisfaction, anxiety, depression, bulimic risk, eating disorders, and perceived stress in adolescents with polycystic ovary syndrome (PCOS). * As showed in the figure, the Spearman correlation test showed a statistically significant positive correlation between body image distress with state and trait anxiety, depression, bulimic risk, eating disorders, and stress.

The Spearman correlation test showed a statistically significant positive correlation between body image distress with weight (rho = 0.423, *p* < 0.001), BMI (rho = 0.499, *p* < 0.001), WC (rho = 0.373, *p* < 0.001), insulin (rho = 0.253, *p* = 0.01), HOMA index (rho = 0.24, *p* = 0.02), VAI (rho = 0.205, *p* = 0.03), total cholesterol (rho = 0.237, *p* = 0.01), LDL (rho = 0.241, *p* = 0.006), and triglycerides (rho = 0.22, *p* = 0.02). Furthermore, body image distress was positively correlated with state (rho = 0.328, *p* < 0.001) and trait (r = 0.447, *p* < 0.001) anxiety, depression (r = 0.397, *p* < 0.001), bulimic risk (r = 0.530, *p* < 0.001), eating disorders (r = 0.514, *p* < 0.001), and stress (r = 0.359, *p* < 0.001) (Figure 2). No significant correlation was found between body image distress and age, gynecological age, birth weight, blood pressure, glycemia, WHR, AST, ALT, LH, FSH, and E_2_ and androgen levels. 

**Figure 3:**
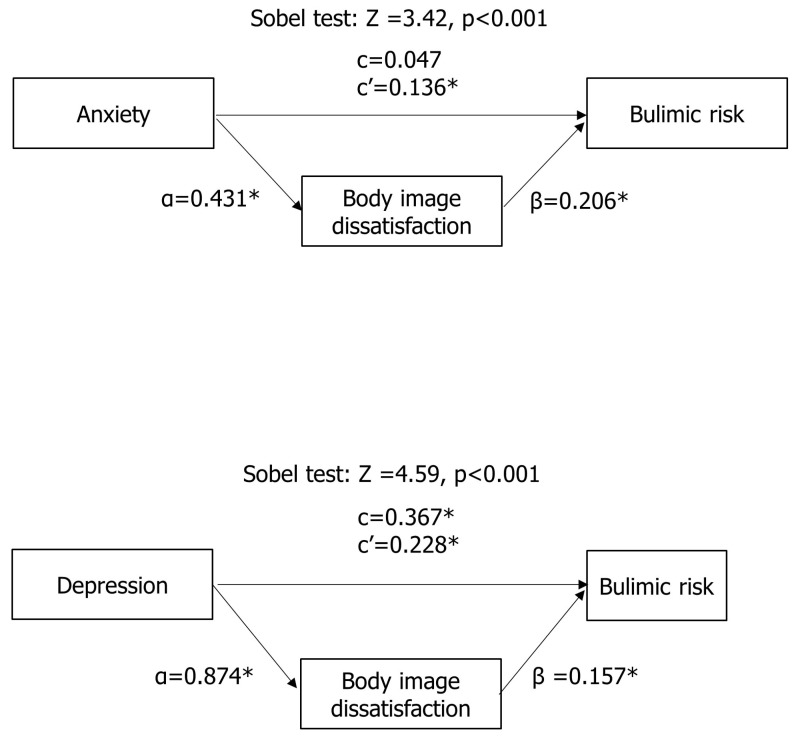
Results from the Sobel test for mediation. Illustration of Sobel coefficients for the mediation of body image dissatisfaction in the relationship between anxiety and bulimic risk and between depression and bulimic risk. The coefficients α, β, and c are Sobel coefficients. Specifically, α = direct effect of the independent variable on mediator; β = effect of the mediator on the dependent variable; c = direct effect of the independent variable on the dependent variable; and c′ = total effect of the independent variable on the dependent variable. * The figure shows that body image dissatisfaction mediated the relationship between anxiety and bulimic risk and between depression and bulimic risk in adolescents with PCOS.

Finally, the Sobel test for mediation showed that body image dissatisfaction mediated the relationship between anxiety and bulimic risk (Z = 3.42, *p* < 0.001) and between depression and bulimic risk (Z = 4.59, *p* < 0.001) in adolescents with PCOS (Figure 3).

## 4. Discussion

Our data showed a high prevalence of psychological vulnerability among adolescents with PCOS. Anxiety was found to be the most prevalent mood disorder in this cohort, with a moderately elevated prevalence of body image dissatisfaction, bulimic risk, depression, and perceived stress. We also found that overweight and IR significantly compromise body image perception in adolescents with PCOS. Body image dissatisfaction during the period of adolescence in a woman’s life may act as a mediating factor in the link between anxious and depressive symptoms and the risk of developing eating disorders.

A recent systematic review and meta-analysis, including 3050 adult women with PCOS and 3858 controls, reported that the prevalence of clinically significant depressive symptoms in women with PCOS was 36.6% versus 15.2% in controls, and the prevalence of anxiety symptoms was 41.9% compared to 8.5% in controls [20]. In the literature, most studies that investigated psychiatric disorders in PCOS were conducted on adult populations, and these results may not reflect an adolescent population. Çoban et al. reported a higher prevalence of psychiatric diagnosis in 28 adolescents with PCOS (aged 13–18 years) compared to age- and sex-matched healthy controls [21]. The recent 2023 International Guideline for PCOS recommend that clinicians screen all adolescents and adults for anxiety and depression using validated screening tools [1]. However, the recognition of mood disorders, particularly during adolescence, remains notably underestimated in PCOS.

The etiology of symptoms of depression and anxiety in women with PCOS is unclear. It cannot necessarily be accounted for by overweight or distressing PCOS symptoms; however, a role for hyperandrogenism, obesity, and hyperinsulinemia has been suggested. Women with PCOS and lower BMI tend to have slightly lower anxiety and depression scores [22]. This is in line with our findings, which showed a high prevalence of psychological vulnerability among adolescents with PCOS, regardless of BMI. However, adolescent girls with PCOS with overweight and/or IR appear to be at increased risk for body image dissatisfaction and anxiety disorders. The pivotal role of obesity in the pathogenesis of PCOS during adolescence was reported by Ibáñez and Zegher [23]. According to the authors, PCOS should be seen as a reversible, endocrine-metabolic mode, essentially driven by ectopic fat, which, in turn, often results from a mismatch between early adipogenesis and later lipogenesis or between prenatal and postnatal weight gain [23]. 

Furthermore, women diagnosed with PCOS show heightened activity in both the sympathetic nervous system and the hypothalamic–pituitary–adrenal (HPA) axis in reaction to stressors, leading to elevated cortisol levels [24]. It is well known that depression is related to increased cortisol secretion [25]. Hyperactivation of the HPA axis may also play a role in the accumulation of visceral fat and subsequent chronic inflammation, which are typical features of PCOS [24]. Evidence indicates that inflammation can impact mood, facilitating the infiltration of inflammatory cells into the brain. Within the central nervous system (CNS), a heightened production of inflammatory cytokines may influence mood by altering the levels of neurotransmitters implicated in the development of depression [26]. 

A recent cross-sectional study of 189 adult women with PCOS and 225 controls (*n* = 225) showed that the association between PCOS, anxiety, and depression can be mediated by body image distress [27]. PCOS can have a profound impact on body image and self-esteem, as patients may struggle with weight gain and signs of hyperandrogenism. In our cohort, we found that obesity and IR significantly impaired body image perception, which, in turn, may mediate the relationship between anxiety and depressive symptoms with bulimic risk. The way people perceive and feel their bodies, especially during the critical phase of adolescence, can play a fundamental role in influencing the manifestation of mental health problems. Many patients with PCOS report frustration with the inability to lose weight, low self-esteem, and, consequently, poor body image [28]. Cultural influences are likely involved in body image distress, as the android fat distribution commonly associated with PCOS may be considered unattractive. Moreover, social expectations of thinness in Western countries may also play a role. This has worsened in the digital age, where adolescents have greater access to social media and are constantly comparing themselves to others and to unrealistic role models on various social media platforms [29]. Social expectations, promoted by social media, have been related to the onset of risk factors for eating disorders, especially during adolescence [30]. Menstrual irregularities due to different mechanisms are strongly associated with the presence of eating disorders [31]. While anorexia is highly prevalent in hypogonadal women, binge eating disorders are found more in amenorrheic women with hyperandrogenism [32]. 

Previous studies have suggested that women with PCOS may be prone to binge eating [33] and bulimia nervosa [34,35]. Of note, androgens can trigger binge-purging behaviors [36]. Furthermore, reproductive steroids exert a profound impact on neuroendocrine pathways in the female brain, modulating a vast array of neurotransmitters and neuropeptides that play a pivotal role in many emotional and behavioral responses [37]. At the same time, binging on high-fat, high-calorie foods contributes to a decreased insulin sensitivity, and it increases fasting blood glucose and insulin levels [38]. In turn, hyperinsulinemia stimulates the synthesis of ovarian androgens, promoting a vicious cycle that exacerbates PCOS symptoms and the progression of metabolic disorders [20]. Insulin receptors are located in the brain, particularly in areas that regulate the brain’s response to appetite stimuli, as well as homeostatic and hedonic food intake [38]. Neuroimaging studies have revealed that women with PCOS, particularly those with IR, show greater activation in regions associated with reward in response to food cues. Furthermore, they show an inability for glucose to suppress this activation, potentially leading to non-homeostatic eating behaviors and a propensity for weight gain and obesity [39]. In addition, obese women with PCOS showed decreased global brain volumes compared with BMI-matched brain controls, suggesting a more evident disruption to the brain structure when PCOS is accompanied with obesity. Suboptimal brain functioning seen in women with PCOS may be related with decreased psychological well-being [39]. Patients with PCOS tend to underestimate their total food intake, especially foods rich in simple sugars [40]. This behavior may reflect eating disorders, of which PCOS patients are still unaware during adolescence. Underestimation of the energy intake has been positively correlated with overweight/obesity and negative body image perception in both adolescents and adults [41,42].

Therefore, the study of these aspects during adolescence becomes crucial to understanding the progression of the syndrome and promoting emotional balance and adherence to lifestyle change programs, which represent the first-line treatment in PCOS [43]. Moreover, women with PCOS, compared to women without, often report greater difficulty achieving weight loss [43]. Addressing psychological issues in PCOS patients not only improves mood but also promotes adherence to a healthier lifestyle and reduces biological factors (such as inflammation and hyperandrogenism), which contribute to long-term complications [21]. In women with PCOS and comorbid mood disorders, combining cognitive behavioral therapy (CBT) with lifestyle modifications leads to significantly greater weight loss than lifestyle changes alone [21]. A bidirectional relationship links lifestyle changes and psychological health: women who are psychologically healthier are more likely to sustain a healthy lifestyle; on the other hand, lifestyle changes can significantly contribute to a positive mood [24].

In addition, eating and mood disorders are a subject of considerable interest in the context of PCOS [44]. In a recent review, Steegers-Theunissen et al. hypothesized that PCOS itself could be considered a brain disorder induced by psychological distress and episodes of overeating and/or dieting during puberty and adolescence [45]. The authors proposed that psychological stressors (such as stressful family environment, difficulty with homework, bullying) associated with eating disorders, which are common during adolescence, may epigenetically upregulate the activation of the central hypothalamic–pituitary–gonadal axis, and, in turn, contribute to the development of PCOS [45]. The incidence of eating disorders in girls with PCOS is increased in overweight and obese subjects, which, in turn, may serve to further exacerbate the condition. This is in accordance with our results, suggesting there is a need for greater attention to adolescent patients with PCOS and metabolic features. 

This study has some limitations. First, the study has a cross-sectional design, which, therefore, limits the interpretation of the causal association between mental health and the typical characteristics of PCOS. The lack of a matched population control group is a relevant limitation, as well as the availability of circulating androgens measured with standard assays. Another limitation is the lack of scores from standardized visual scales, such as the modified Ferriman–Gallwey score for the assessment of hirsutism. The strength of the present study is that it was conducted using validated instruments, encompassing several psychological dimensions in a well-characterized sample of Italian adolescents with PCOS. Future prospective randomized studies to assess the role of therapeutic interventions in enhancing mental well-being of adolescents with PCOS are needed.

## 5. Conclusions

The results of this study showed that a considerable number of patients with PCOS have a psychological vulnerability in adolescence. Our findings also highlight the impact of obesity and IR on the body image of young PCOS patients, potentially leading to the development of further psychological issues, including eating disorders. Raising awareness and prioritizing mental health screening in PCOS patients are vital measures to recognize the often under-reported psychological aspects associated with this syndrome. A comprehensive biopsychosocial approach in adolescents with PCOS represents the basis for effectively managing and preventing complications resulting from both psychological and biological disorders in adulthood. 

## Figures and Tables

**Table 1:** Demographic, clinical, and metabolic characteristics of adolescents with polycystic ovarian syndrome (PCOS) (menstrual irregularities + hyperandrogenism).

	PCOS Adolescents (*n* = 128)	
	Mean ± SD	Median (IQR)
**Demographic characterstics**		
Age (years)	17.2 ± 1.6	17 (16–19)
Age at menarche (years)	11.9 ± 1.3	12 (11–13)
Gynecological age (years)	5.2 ± 1.8	5 (4–7)
Education (years)	11.2 ± 1.6	11 (10–13)
**Clinical characteristics**		
Birth weight (g)	3112.7 ± 589.9	3120 (2790–3465)
Weight (kg)	66.4 ± 16.9	62 (55.1–70.4)
Body mass index (kg/m^2^)	24.8 ± 6.2	23.1 (20.4–27.6)
Waist circumference (cm)	79.7 ± 14.1	77 (70–85)
Waist/hip ratio	0.8 ± 0.07	0.8 (0.75–0.84)
Systolic blood pressure (mmHg)	111.7 ± 14.6	110 (105–120)
Diastolic blood pressure (mmHg)	70.7 ± 8.3	70 (65–75)
**Metabolic profile**		
Fasting glycemia (mg/dL)	81.2 ± 8.1	70 (65–70)
Fasting insulin (mIU/mL)	11.5 ± 9.3	9.2 (6.2–14.8)
HOMA index	2.4 ± 2.06	1.8 (1.3–3.01)
Visceral adiposity index	2.6 ± 1.7	2.2 (1.4–3.2)
Total cholesterol (mg/dL)	152.4 ± 30.9	149 (133–174)
High-density lipoprotein cholesterol (mg/dL)	52 ± 12.7	51 (43–58)
Low-density lipoprotein cholesterol (mg/dL)	77.8 ± 35.2	80.4 (62.3–101.2)
Triglycerides (mg/dL)	69.7 ± 36.9	58 (44–89)
Aspartate transaminase (mU/mL)	19.7 ± 9.9	18 (15–20)
Alanine transaminase (mU/mL)	20.5 ± 19.7	15 (13–19.8)
**Hormonal profile**		
Luteinizing hormone (LH) (IU/L)	8.9 ± 5.6	7.3 (5.2–11.2)
Follicle-stimulating hormone (FSH) (IU/L)	5.3 ± 1.8	5.2 (4.1–6.1)
LH/FSH ratio	1.7 ± 0.9	1.5 (1–2.2)
17β-estradiol (pg/mL)	61.1 ± 51.2	46.5 (33.9–63.5)
Androstenedione (ng/mL)	4.1 ± 2	3.7 (2.7–4.8)
Total Testosterone (ng/mL)	0.7 ± 0.4	0.7 (0.4–1)
DHEAS (ng/mL)	3.3 ± 2.8	2.7 (2–3.6)

**Table 2:** Psychometric characteristics in adolescent girls with polycystic ovarian syndrome (PCOS).

	PCOS Adolescents (*n* = 128)	
	Median of Total Score (IQR)	Percentage of Patients with a Pathological Score (%)
Body attitude (BAT)	35 (26–48)	49.2% (*n* = 63/128)
State anxiety (STAI-I)	43 (36–51.8)	57% (*n* = 73/128)
Trait anxiety (STAI-2)	44.5 (36.2–53)	63.1% (*n* = 81/128)
Depression (BDI)	8 (3.2–12)	39.1% (*n* = 50/128)
Bulimia (BITE)	9 (6–14)	41.4% (*n* = 53/128)
Eating disorder (EAT)	7 (3–13)	11.7% (*n* = 15/128)
Perceived stress (PSS)	21 (18–25)	68.2% (*n* = 60/88)

**Table 3:** Comparison of psychometric characteristics in adolescents with polycystic ovary syndrome (PCOS) according to body mass index (BMI) and insulin resistance (IR).

	Body Mass Index (BMI)	*p*-Value	Insulin Resistance (IR)	*p*-Value	BMI & IR	*p*-Value
	Median of Total Score (IQR)		Median of Total Score (IQR)		Median of Total Score(IQR)	
Body attitude (BAT)	**<18.5 (*n* = 9)** 20 (17.5–29) **18.5–24.9 (*n* = 76)** 32 (23.25–46.2) **25–29.9 (*n* = 19)** 38 (32–44) **>30 (*n* = 24)** 52.5 (40.2–64.7)	**<0.001**	**IR (*n* = 30)** 47 (32.5–58) **NO IR (*n* = 98)** 32 (24–40)	**0.003**	**BMI < 25 + HOMA < 2.5 (*n* = 73)** 20 (17.5–29) **BMI ≥ 25 + HOMA < 2.5 (*n* = 27)** 32 (23.25–46.25) **BMI < 25 + HOMA ≥ 2.5 (*n* = 5)** 38 (32–44) **BMI ≥ 25 + HOMA ≥ 2.5 (*n* = 23)** 52.50 (40.25–64.75)	***p* < 0.001**
State anxiety (STAI-I)	**<18.5 (*n* = 9)** 42 (30.5–52) **18.5–24.9 (*n* = 76)** 42.5 (36.25–50) **25–29.9 (*n* = 19)** 38 (35–50) **>30 (*n* = 24)** 47.5 (36.5–57)	0.61	**IR (*n* = 30)** 44 (36–55.5) **NO IR (*n* = 98)** 42 (36–50)	**0.032**	**BMI < 25 + HOMA < 2.5 (*n* = 73)** 42 (30.5–52) **BMI ≥ 25 + HOMA < 2.5 (*n* = 27)** 42.5 (36.25–50) **BMI < 25 + HOMA ≥ 2.5 (*n* = 5)** 38 (35–50) **BMI ≥ 25 + HOMA ≥ 2.5 (*n* = 23)** 47.5 0 (36.50–57.50)	0.085
Trait anxiety (STAI-2)	**<18.5 (*n* = 9)** 45 (37–52) **18.5–24.9 (*n* = 76)** 44.5 (37–51.75) **25–29.9 (*n* = 19)** 41 (34–50) **>30 (*n* = 24)** 49.5 (36–55.7)	0.65	**IR (*n* = 30)** 45 (36–53) **NO IR (*n* = 98)** 44 (37–53)	0.10	**BMI < 25 + HOMA < 2.5 (*n* = 73)** 45 (37–52) **BMI ≥ 25 + HOMA < 2.5 (*n* = 27)** 44.50 (37–51.75) **BMI < 25 + HOMA ≥ 2.5 (*n* = 5)** 41 (34–50) **BMI ≥ 25 + HOMA ≥ 2.5 (*n* = 23)** 49.5 (36–55.75)	0.458
Depression (BDI)	**<18.5 (*n* = 9)** 9 (5–18) **18.5–24.9 (*n* = 76)** 7.5 (4–11.75) **25–29.9 (*n* = 19)** 8 (4–12) **>30 (*n* = 24)** 8 (2–14.75)	0.80	**IR (*n* = 30)** 8 (4–12.5) **NO IR (*n* = 98)** 7 (3–12)	0.21	**BMI < 25 + HOMA < 2.5 (*n* = 73)** 9 (5–18) **BMI ≥ 25+HOMA < 2.5 (*n* = 27)** 7.5 (4–11.75) **BMI < 25 + HOMA ≥ 2.5 (*n* = 5)** 8 (4–12) **BMI ≥ 25 + HOMA≥2.5 (*n* = 23)** 8 (2–14.75)	0.544
Bulimia (BITE)	**<18.5 (*n* = 9)** 7 (6–12.5) **18.5–24.9 (*n* = 76)** 8 (5–14) **25–29.9 (*n* = 19)** 9 (6–12) **>30 (*n* = 24)** 11 (6–14.75)	0.74	**IR (*n* = 30)** 9 (6–14) **NO IR (*n* = 98)** 8.5 (5.7–13.25)	0.83	**BMI < 25 + HOMA < 2.5 (*n* = 73)** 7 (6–12-5) **BMI ≥ 25 + HOMA < 2.5 (*n* = 27)** 8 (5–14) **BMI < 25 + HOMA ≥ 2.5 (*n* = 5)** 9 (6–12) **BMI ≥ 25 + HOMA ≥ 2.5 (*n* = 23)** 11 (6–14.75)	0.815
Eating disorder (EAT)	**<18.5 (*n* = 9)** 4 (3–9.5) **18.5–24.9 (*n* = 76)** 6.5 (3–13.5) **25–29.9 (*n* = 19)** 6.5 (3.7–12) **>30 (*n* = 24)** 10 (5.7–16.5)	0.20	**IR (*n* = 30)** 7 (4–13.5) **NO IR (*n* = 98)** 7 (3–13)	0.70	**BMI < 25 + HOMA < 2.5 (*n* = 73)** 4 (3–9.5) **BMI ≥ 25 + HOMA < 2.5 (*n* = 27)** 6.5 (3–13.5) **BMI < 25 + HOMA ≥ 2.5 (*n* = 5)** 6.5 (3.75–12) **BMI ≥ 25 + HOMA ≥ 2.5 (*n* = 23)** 10 (5.75–16.5)	0.841
Perceived stress (PSS)	**<18.5 (*n* = 9)** 20 (18–23) **18.5–24.9 (*n* = 76)** 22 (16–25.25) **25–29.9 (*n* = 19)** 21 (14.2–27) **>30 (*n* = 24)** 21 (19–25)	0.72	**IR (*n* = 30)** 21 (18–25) **NO IR (*n* = 98)** 21 (17–25)	**0.035**	**BMI < 25 + HOMA < 2.5 (*n* = 73)** 20 (18–23) **BMI ≥ 25 + HOMA < 2.5 (*n* = 27)** 22 (16–25.25) **BMI < 25 + HOMA ≥ 2.5 (*n* = 5)** 21 (14.25–27) **BMI ≥ 25 + HOMA ≥ 2.5 (*n* = 23)** 21 (19–25)	0.329

Abbreviations: HOMA, homeostasis model assessment index.

## Data Availability

The data presented in this study are available on request from the corresponding author. The data are not publicly available due to ethical restriction.

## Supplementary Materials

The following supporting information can be downloaded at: https://www.mdpi.com/article/10.3390/children11050521/s1, Table S1: Comparison of body attitude factors in adolescents with polycystic ovary syndrome according to the body mass index (BMI) and insulin resistance.
