# Supplementary material for: Body Image and Other Mood Vulnerabilities in Adolescents with Polycystic Ovary Syndrome and Metabolic Alterations
Source: Children (Basel). 2024 Apr 26;11(5):521. doi: 10.3390/children11050521 (PMC11119722; doi:10.3390/children11050521)
Supplement: Supplementary file 1 [file children-11-00521-s001.zip › children-2955759-supplementary.pdf]

Supplementary table S1. Comparison of body attitude factors in adolescents with polycystic ovary syndrome according to the body mass index (BMI) and insulin resistance.

Body attitude factors	BMI & Insulin resistance	Median (95% CI)	p-Value
Negative appreciation of body size	**BMI <25 + HOMA <2.5 (n=73)**	7 (3-15)	<0.001
	**BMI ≥25 + HOMA <2.5 (n=27)**	16 (13-23)	
	**BMI <25 + HOMA ≥2.5 (n=5)**	8 (3-27)	
	**BMI ≥25 + HOMA ≥2.5 (n=23)**	19 (15-23)	
Lack of familiarity with one's own body	**BMI <25 + HOMA <2.5 (n=73)**	9 (7-10)	0.03
	**BMI ≥25 + HOMA <2.5 (n=27)**	9 (7-12)	
	**BMI <25 + HOMA ≥2.5 (n=5)**	10 (5-15)	
	**BMI ≥25 + HOMA ≥2.5 (n=23)**	12 (10-15)	
General body dissatisfaction	**BMI <25 + HOMA <2.5 (n=73)**	7 (4-10)	<0.001
	**BMI ≥25 + HOMA <2.5 (n=27)**	11 (9-15)	
	**BMI <25 + HOMA ≥2.5 (n=5)**	6 (3.5-16)	
	**BMI ≥25 + HOMA ≥2.5 (n=23)**	11 (8-15)	
Rest Factor	**BMI <25 + HOMA <2.5 (n=73)**	7 (5-9)	0.160
	**BMI ≥25 + HOMA <2.5 (n=27)**	7 (4-8)	
	**BMI <25 + HOMA ≥2.5 (n=5)**	8 (4-9.5)	
	**BMI ≥25 + HOMA ≥2.5 (n=23)**	6 (4-7)	

Abbreviations: HOMA, homeostasis model assessment index.
